# The science of YouTube: What factors influence user engagement with online science videos?

**DOI:** 10.1371/journal.pone.0267697

**Published:** 2022-05-25

**Authors:** Shiyu Yang, Dominique Brossard, Dietram A. Scheufele, Michael A. Xenos

**Affiliations:** 1 Department of Life Sciences Communication, University of Wisconsin-Madison, Madison, Wisconsin, United States of America; 2 Morgridge Institute for Research, Madison, Wisconsin, United States of America; Ca’ Foscari University of Venice, ITALY

## Abstract

As the reach of science content in traditional media declines, many institutions and scientists are turning to YouTube as a powerful tool for communicating directly with non-expert publics. They do so with little empirical social science research guiding their efforts. This study explores how video characteristics and social endorsement cues provided by audience members might influence user engagement with online science videos. Shorter videos are more likely to be viewed. Social endorsement cues significantly relate to variations in user engagement, with likes having a consistent positive association with all types of engagement. Implications for science communication through YouTube are discussed.

## Introduction

Research and practice in science communication have a long tradition of focusing on legacy media given their primary role in disseminating news about scientific breakthroughs and bridging the science-public divides. However, nowadays such media no longer have the central place they used to occupy in the science communication process [1,2]. Indeed, individuals are increasingly turning to online sources for scientific information and audiences for traditional print and broadcast media are shrinking especially for science and technology news [1]. In addition, the amount of space devoted to science and technology news (e.g., news time on television, column inches in print newspapers) in traditional media outlets is dwindling. Science journalists, traditionally in charge of translating complex scientific information into formats that lay audiences find interesting and can easily digest, are vanishing from traditional newsrooms [2].

Faced with the deteriorated influence of legacy media in science communication, many leaders in the scientific community have called for scientists to engage and communicate directly with the public [3,4] and argued that there is an urgent need to forge more responsive and closer connections between the scientific community and the general population as society moves forward with complex science developments [3]. New communication tools give members of the scientific community the opportunity to directly engage with lay audiences. Online communication channels have significantly reshaped how people seek and understand scientific information [5] and have blurred boundaries between scientists, science journalists, and general audiences [6]. The new media information environment is by nature “pluralistic, participatory and social” [6] and presents unique opportunities for engaging the wider public with science.

The video-sharing site YouTube is particularly well suited for successful science communication online. As the world’s largest video-sharing site [7], YouTube has over 1.5 billion monthly active users, which amount to approximately one-third of Internet users [8]. Among the top categories of YouTube videos (with views and subscribers), science and technology videos generate a total of 12.2 billion monthly views, attract 1.3 billion subscribers, and are viewed for a total of 766.7 million hours [9]. Given YouTube’s exceptionally far reach and potentially impactful role in disseminating scientific information, there has been an increasing interest in using its platform for science outreach purposes [10–12]. The small but growing body of empirical evidence guiding the practice of communicating science through YouTube reflects this increasing interest among communication scholars and practitioners [13].

Within this context, we provide initial insights on the makeup of YouTube science audiences and some of the factors that influence user engagement with YouTube science videos, by analyzing a unique, comprehensive set of back-end data from YouTube Analytics made available by the American Chemical Society. While previous research on YouTube and science communication has almost exclusively focused on the relationship between video content factors and user engagement, we explore how video characteristics that are not primarily content-related, including audience cues, associate with variations in user engagement. Such non-primarily content-related factors are important to understand because they reflect the larger infrastructural and algorithmic influence of the YouTube platform, which plays a central role in shaping video popularity but remains a black box to outsiders. Moreover, we expand current understanding of user engagement on YouTube by exploring additional engagement activities that are, albeit understudied, useful for garnering a fuller understanding of user engagement on YouTube. By showing how different video characteristics and audience cues can influence a variety of user engagement activities with online science videos, our study demonstrates the importance of attending to these factors in using YouTube for science communication and sheds light on specific strategies that may make science videos more likely to cut through the online noise and effectively reach viewers.

## Literature review

### Outcome of interest: User engagement with YouTube videos

Our primary outcome variable is user engagement with science videos on YouTube. In the broadest term, user engagement speaks to user-initiated actions that contribute to co-creation of value and knowledge among the online community [14,15]. This definition echoes Xenos et al.s’ conceptualization of user response to social media campaigning which describes user engagement as “observable activities” that are directly linked to specific “communications within social media” and create a resource in themselves as well as signal other resources [16]. In our case, user engagement activities are directly linked to science videos on YouTube. Moreover, user engagement “places a sharper focus on” click- and comment-based interactions with specific digital objects, such as liking and commenting on a social media post [16]. In the YouTube context, this may include viewing, liking, disliking, sharing and commenting on a video post.

Research on user engagement with YouTube videos has traditionally focused on popularity metrics such as video views, comments, (dis)likes [17–21] and less commonly, shares [22]. Some have also argued for including comment-reading and video-uploading as forms of user engagement [23]. Building on this line of work, we expand the current understanding of user engagement on YouTube by considering additional engagement phenomena besides traditional click- and comment-based activities, such as average view duration of a video, average percentage of a video being viewed, number of subscribers gained by a video, and number of playlists a video is added in.

The additional measures that we propose complement existing measures of user engagement in a number of ways: first, unlike click- and comment-based interactions, average view duration and average percentage viewed reveal closer details about the way users consume a video, since these two measures track down how well the video holds the attention of the users once they are pulled in; second, a video may intrigue users so much that they want to see more videos from the channel in the long term, by subscribing to the channel after watching a video, which signifies a deeper level of commitment and engagement than one-time clicks and comments; third, users can save a video in playlists for future reference if they are not able to watch it immediately upon encountering it despite finding it interesting, or if they find the video so relevant that they want to come back to it again in the future. Despite the distinct value of understanding these forms of user engagement, these phenomena are rarely studied by prior research because they typically require access to back-end YouTube data, which can be quite unobtainable. By exploring these additional user activities, we expand the scope of user engagement currently defined and capture a fuller picture of user engagement with science videos on YouTube.

### YouTube: Audiences, science videos, and mechanism

Founded in 2005 and purchased by Google in 2006, YouTube has grown to be the second largest network channel in the world [24] and the third most visited site after Google.com and Facebook.com [25]. Every eight out of ten 18- to 49-year-olds watch YouTube videos monthly [26] and 122 million active users are attracted to YouTube daily [27]. Among all YouTube users, 62% are males and 38% are females [8]. The 25–44 age group watch the most YouTube videos [8]. Within the United States, YouTube is the most widely used online platform among US adults [28]. Nearly three-quarters of the adult population and 91% of 18- to 29-year-olds use YouTube, with 51% of users reporting visiting the site daily [29].

Science and technology is one of the most prominent video categories on YouTube, accounting for approximately 4% of all video uploads and ranking seventh of all categories in 2017 [8,30]. Some of the most popular science-themed channels enjoy tens of millions of subscribers and billions of views and are viewed tens of millions of times every month [31,32]. There are not a lot of statistics on how many scientists are on YouTube, however. An online survey asking 587 scientists what types of social media services they used found that use of YouTube among scientists was infrequent, although this might have to do with confusion over the definition of social media services [33]. A 2015 survey of a convenience sample of 233 scientists showed that nearly half of surveyed scientists used media sharing sites such as YouTube and Flickr at least weekly [34]. Another survey of scientists from an American R1 University revealed that 43% of surveyed scientists used YouTube for “science-related purposes” at least a few times a month [35]. There is also a lack of data on the makeup of audiences for YouTube science videos specifically.

With the aim of maximizing user retention over the long run [36], YouTube adopts a recommendation system that suggests potentially relevant videos to users out of an ever-growing massive collection of videos [37]. The YouTube recommendation system determines the order in which a video shows up in search results, suggested related videos, YouTube home page, and the like [36,38] and is a primary source of video views [39]. Needless to say, the more a video is recommended by YouTube, the greater chances users will engage with the video. In an analysis examining different sources of views, Zhou et al. found that YouTube search and related video recommendation are the two major sources that drive video views and stabilize view rate. Moreover, related video recommendation was found to help audiences find niche videos while YouTube search and video highlight drive viewers to videos that are already popular [7].

The YouTube recommendation system takes into account both video content-based and content-independent factors when making recommendations [40]. While much of the research studying user engagement with science videos on YouTube has almost exclusively focused on the impact of content factors, factors that are not primarily content-related play an equally, if not more, important role in influencing video popularity, although the mechanism of their influence is not directly observable [18]. Existing literature suggests that several video characteristics including number of views, likes, shares, and comments appear to affect how likely a video is to be recommended by YouTube [9,38]. Videos with more views, likes, shares, and comments are more likely to be recommended to users than videos that score low on those indicators [41], as those indicators signal video quality [38]. Video view count, comments, and likes have also been found to positively correlate with one another [19]. In addition, longer videos are more likely to be recommended than shorter videos, perhaps because longer videos generate greater watch time [9,42].

Many of the factors influencing YouTube recommendations (e.g., video views, likes, comments) are visible to YouTube users and provide social information about the video; that is, they may serve as cues of social endorsement and carry their own psychological implications for stimulating user engagement. In the next section, we review how social endorsement cues may shape user engagement with online information.

### Social endorsement cues and online science communication

Users can derive from social endorsement cues the knowledge about how likely a piece of online information is to be correct or good, since these cues are indicative of other users’ (dis)approval of the information [43]. Endorsement cues also signal how relevant or interesting the information will be to us personally because we tend to “assume that the support of others is likely to predict personal relevance and utility” [44]. In other words, social endorsement cues provide users with cognitive shortcuts to reaching judgments about online information [43]. Cues of endorsement on online information from other users can be especially powerful at influencing perceptions [45,46] and guiding user behaviors [44,47–50].

Employing an experimental design in which participants watched one of two science comedy clips, Cacciatore et al. found that audience laughter included in the video clip could serve as a powerful social cue indicating others’ endorsement on the content, which increased participants’ favorable affective response to the video clip and their intentions to engage with science more broadly [51]. In an artificial music market, Salganik and colleagues found that showing participants social cues that were indicative of the popularity of songs (e.g., number of times a song had been downloaded by other users, ranking of songs presumably based on popularity) significantly influenced users’ subsequent downloading behavior of songs [49]. Cues of endorsement also influence users’ attention to online information [52]. On a mock news website, articles marked with a high number of Facebook likes were selected by users more often and earlier, and were read for longer than articles accompanied by a low number of likes [53]. Other researchers have also examined how social endorsement cues embedded in the Facebook environment affected news choice [44], voting behavior [47], preventive behavioral intentions [54], and credibility perceptions of health and science information [45,55].

While a plethora of studies have examined how user behaviors may be shaped by social endorsement cues in other social media contexts, few studies have paid attention to the YouTube platform, the second largest social networking space [24]. In their experiment on how video view count may cue YouTube users’ normative perceptions about the salience of the issue of climate change, Spartz et al. found that the climate change video that had a higher number of views, compared with an identical video with much fewer views, led viewers to perceiving greater importance assigned to the issue of climate change by fellow Americans [46]. Although scholars have examined perceptions as a function of social endorsement on YouTube [46], research on how social endorsement cues in the YouTube space may shape user behaviors, particularly user engagement with online videos, is scarce. Our study represents an initial effort to fill in this gap and builds on prior work as we explore how social endorsement cues may relate to user engagement with YouTube science videos, an area deserving greater attention from science communication scholars.

Taken together, we propose the following research question about user engagement with science videos on YouTube, which has particular relevance to practitioners of science communication:

RQ1: To what extent are video characteristics and social endorsement cues embedded in the YouTube environment associated with levels of user engagement with science videos?

## Method

To understand variations in user engagement with YouTube science and chemistry videos, we partnered with the American Chemical Society to analyze comprehensive user data of their YouTube channel *Reactions*. *Reactions* channel is one of the fastest growing science education channels on YouTube that produces short, entertaining videos about everyday chemistry and science. As of the time this paper was written, *Reactions* had gained more than 36 million views and 298,805 subscribers since its launch in January 2014. Our data came from YouTube Analytics and were made available by the American Chemical Society, which provided us with the unique opportunity to access a primary dataset with information from millions of users on aggregate audience characteristics, user engagement, and video characteristics for a total *N* = 210 videos since the channel’s launch to June 9, 2017. Addressing potential concerns about research collaborations between academic and industry partners [56], all data sharing and analyses were approved by the University of Wisconsin-Madison Institutional Review Board (IRB). Informed consent was not obtained because the data were analyzed anonymously.

Before we dive into the dataset and variables, it is worth noting that our methodological approach has several advantages. First, our data recorded behaviors of YouTube users as they interacted with real-world stimuli in natural environments––––rather than artificial manipulation of the independent variables in a lab setting, which oftentimes does not equate with how complex dynamics play out in the real world [57]. In other words, our approach has higher ecological validity than experimentation. Further, although we cannot completely separate effects of video quality from effects of video characteristics and social endorsement cues on user engagement without experimentally testing the causal relationship between social endorsement cues and user engagement, we have reason to believe that effects of video quality on user engagement are minimal due to the fact that all videos in our dataset were professionally produced by a single channel and thus are of similar production quality. Second, focusing on a single channel is advantageous in that it helps rule out potential channel confounds behind video characteristics, audience makeup, and audience engagement [21].

The dataset contained dozens of variables about a variety of video characteristics (e.g., video title, length, creation date, ad revenue, annotations shown, number of cards shown, among others), aggregate information on audience characteristics (e.g., age, gender, country, subscription status, subscription source type, access device, access operating system, traffic source type, use of translation, use of subtitles and closed captioning, sharing service, sharing device, among many others) and user engagement activities (views, average view duration, average percentage viewed, likes, dislikes, shares, comments, subscribing and unsubscribing, adding to and removing from playlists, among others). Note that user engagement data were broken down either by video or by aggregate audience characteristic. User engagement data at the channel level were also available on a day-to-day basis. We draw on these comprehensive data to explore how user engagement with science videos is shaped by algorithmic and social influences.

## Key measures

### Dependent variables: User engagement with YouTube videos

We examine a number of dependent variables that capture aspects of user engagement with YouTube videos, including video view count (Min = 2,470, Max = 1,439,920, Median = 48,203, Mean = 122,574, SD = 198789.60), shares (Min = 3, Max = 4,055, Median = 172, Mean = 296.11, SD = 408.90), comments (Min = 4, Max = 1,383, Median = 48.50, Mean = 92.61, SD = 145.12), average view duration (Min = 0.40 min, Max = 4.10 min, Median = 2.20 min, Mean = 2.20, SD = 0.62 min), average percentage viewed (Min = 21.14%, Max = 82.10%, Median = 64.47%, Mean = 63.62%, SD = 7.84%), number of subscribers gained (Min = 2, Max = 4,989, Median = 96, Mean = 295.53, SD = 608.00), and number of playlists added in (Min = 38, Max = 4,848, Median = 350, Mean = 563.73, SD = 647.45).

### Independent variables

Several video characteristics and social endorsement cues including video length (Min = 0.62 min, Max = 6.55 min, Median = 3.40 min, Mean = 3.50 min, SD = 1.03 min), view count (Min = 2,470, Max = 1,439,920, Median = 48,203, Mean = 122,574, SD = 198789.60), likes (Min = 29, Max = 4,115, Median = 476, Mean = 720.00, SD = 692.19), dislikes (Min = 1, Max = 2,285, Median = 22.5, Mean = 58.29, SD = 176.77), and comments (Min = 4, Max = 1,383, Median = 48.5, Mean = 92.61, SD = 145.12) were included as independent variables in our analysis of user engagement because a) prior work suggests that these factors presumably affect how YouTube makes video recommendations, which subsequently influences user engagement with online science videos; and b) video view count, likes, dislikes, and comments are visible to users as they visit YouTube video webpages and thus serve as social endorsement cues that could impact user psychology relative to engaging with online science videos. Count independent variables including view count, likes, dislikes, and comments were log transformed due to skewness.

### Analytic plan

To contextualize our findings, we first provide summary user engagement data broken down by key demographic and audience characteristic variables in Tables 1 through 4. In addressing RQ1, to explore to what extent video characteristics and social endorsement cues shape user engagement with YouTube science videos, we employ negative binomial regression and hierarchical least ordinary squares regression. Negative binomial regression was the appropriate analytic choice for the analyses of video view count, comments, shares, number of subscribers gained, and number of playlists added in, given that these dependent variables are essentially count data and because of the over-dispersion of the distribution of these five variables where the variance exceeds the mean [16,58,59]. Negative binomial regression enters blocks of variables following presumed causal sequence and allows researchers to test the extent to which the additional block of variables adds to the explanatory power above and beyond the preceding model. We report likelihood-ratio test statistics (i.e., Chi-Square, degrees of freedom, p-value) to test the contribution of each variable block and goodness-of-fit measures (i.e., AIC, BIC) to assess model fit. Hierarchical ordinary least squares (OLS) regression was used in the analyses of average view duration and average percentage viewed due to the continuous nature of these outcome variables. Similar to negative binomial regression, hierarchical OLS regression also allows researchers to assess the relative explanatory power of different independent variables by entering them in blocks based on their presumed causal order.

**Table 1:** Viewership by age and gender.

	Views (% of total views)		Average View Duration (minutes)		Average Percentage Viewed (%)	
	Female	Male	Female	Male	Female	Male
13–17 years	2.00	3.30	1.90	2.10	57.51	61.76
18–24 years	7.80	19.70	2.00	2.20	61.15	65.02
25–34 years	8.00	28.30	2.10	2.20	61.73	66.30
35–44 years	3.60	11.30	2.00	2.20	61.25	65.21
45–54 years	2.40	5.40	2.10	2.20	64.61	66.02
55–64 years	1.60	2.90	2.20	2.30	67.28	67.90
65+ years	1.10	2.60	2.10	2.20	67.40	67.54

## Results

Before addressing RQ1, we provide data on audience demographics to shed light on who watches *Reactions* science videos and what their engagement styles are. Overall, younger and male users were the most active audiences of *Reactions* (Table 1). While users of all ages did not seem to differ in the time they spent watching each individual video (i.e., average view duration for single video was approximately 2.20 minutes across all age groups), older users (55- to 64-year-olds and those above 65 years old) tended to watch a greater proportion of each video. Although the majority of views came from non-subscribers of the channel, indicating the substantive reach of the videos beyond channel subscribers, channel subscribers were overall more engaged with the videos (Table 2). Computer and mobile phone were the two most common devices that YouTube users used to watch *Reactions* videos, accounting for approximately 81.32% of total views (Table 3). Among the known access devices, users who watched through mobile phones generated the most likes, shares, as well as dislikes per view of video compared with users watching through the other types of devices. Finally, *Reactions* watchers came from almost all countries and areas across the world, with the U.S., the U.K., Canada, India, and Australia being the top five countries generating the most views (Table 4). About 40.47% of subscribers came from the U.S. and American users were the most active and engaged audiences.

**Table 2:** User engagement by subscription status.

	Subscribed	Not Subscribed
Views	1,989,560	23,778,229
Average View Duration (minutes)	2.50	2.00
Average Percentage Viewed (%)	73.17	61.47
Likes per 1,000 Views	39.90	3.05
Dislikes per 1,000 Views	1.04	.43
Shares per 1,000 Views	6.33	2.09
In Playlists per 1,000 Views	18.51	3.44

**Table 3:** User engagement by access device type.

	Computer	Mobile Phone	Tablet	Game Console	TV	Unknown
Views	10,527,375	10,427,974	3,238,890	758,344	563,154	252,052
Average View Duration (minutes)	2.10	2.00	2.00	2.40	2.50	2.10
Average Percentage Viewed (%)	64.01	59.88	60.15	72.85	75.91	66.87
Likes per 1,000 Views	2.02	2.37	1.47	.52	.45	398.70
Dislikes per 1,000 Views	.11	.43	.16	.02	.03	24.11
Shares per 1,000 Views	.82	1.89	.24	.00	.00	131.84

**Table 4:** User engagement by geography (top 5 view count countries).

	United States	United Kingdom	Canada	India	Australia
Views	12,251,908 (47.55%)	1,359,804 (5.28%)	1,253,040 (4.86%)	788,655 (3.06%)	612,121 (2.38%)
Average View Duration (minutes)	2.3	2.1	2.3	1.6	2.3
Average Percentage Viewed (%)	68.99	63.05	68.97	46.12	68.72
Comments	10,706 (54.61%)	973 (4.96%)	1,041 (5.31%)	348 (1.78%)	383 (1.95%)
Likes	71,515 (47.08%)	7,132 (4.70%)	7,022 (4.62%)	4,823 (3.18%)	3,373 (2.22%)
Dislikes	4,416 (35.98%)	623 (5.08%)	470 (3.83%)	727 (5.92%)	223 (1.82%)
Shares	30,007 (48.17%)	2,137 (3.43%)	2,922 (4.69%)	2,649 (4.25%)	1,023 (1.64%)
In Playlists	48,559 (40.91%)	4,796 (4.04%)	6,446 (5.43%)	4,692 (3.95%)	2,301 (1.94%)
Subscribers	82,733 (40.47%)	9,483 (4.64%)	9,287 (4.54%)	13,483 (6.60%)	4,979 (2.44%)

To answer RQ1, we now turn to negative binomial regression analyses of video view count, shares, comments, number of subscribers gained, and number of playlists added in. Results from the final models are shown in Table 5. In the analysis of viewing behavior, our baseline model included only the control variable, time elapsed since a video was uploaded. Next, we incorporated a block on video characteristics that contained the video length variable. This block addition did not significantly improve the baseline model (*χ*^*2*^ = 2.39, *df* = 1, *p* = .12). The Akaike Information Criterion (AIC) dropped by 0.4 (from 5266.00 to 5265.61) while the Bayesian Information Criterion (BIC) increased by 3 (from 5276.04 to 5278.99). The rules of thumb for interpreting change in the AIC and BIC can be found in Fabozzi et al. [60]. Although we did not find additional and distinct influence of the video length variable over the control variable, we kept video length within our models because it has theoretical relevance to understanding viewer response. The third and final model accounted for social endorsement cues (i.e., likes, dislikes, comments) and explained a significant amount of variance in viewing behavior in addition to the second model (*χ*^*2*^ = 371.50, *df* = 3, *p* < .001). Turning to the fit statistics, both the AIC and the BIC dropped considerably (AIC decrease by 365.5 from 5265.61 to 4900.11; BIC decrease by 355.5 from 5278.99 to 4923.54).

**Table 5:** Correlates of variations in science video views, shares, comments, subscribers gained, and playlists added in (*N* = 210).

	Views	Comments	Shares	Subscribers	In Playlists
*Control variable*					
Time elapsed since upload (in 30 days)	.03 (.00)***	.03 (.00)***	-.01 (.00)***	.06 (.00)***	-.02 (.00)***
*Video characteristics*					
Video length (minute)	-.17 (.03)***	-.00 (.04)	.06 (.04)	-.00 (.03)	.02 (.03)
*Social endorsement*					
Likes (log transformed)	2.42 (.19)***	1.81 (.24)***	1.22 (.26)***	2.35 (.25)***	1.05 (.21)***
Dislikes (log transformed)	.41 (.10)	.51 (.10)***	.14 (.11)	-.50 (.10)***	-.09 (.09)
Comments (log transformed)	.11 (.16)		.12 (.17)	.20 (.16)	.51 (.13)***
Views (log transformed)		-.23 (.16)	.88 (.16)***	.81 (.15)***	.59 (.13)***
*Intercept*	3.27 (.37)***	-1.28 (.41)**	-2.32 (.43)***	-6.89 (.42)***	.12 (.34)

*Note*: Cell entries are negative binomial regression coefficient estimates. Standard errors appear in parentheses. Dispersion parameter for “Views” model = 4.14. Dispersion parameter for “Comments” model = 4.31. Dispersion parameter for “Shares” model = 4.23. Dispersion parameter for “Subscribers” model = 4.88. Dispersion parameter for “In Playlists” model = 6.40.

Significance key:

*p < .05

**p < .01

***p < .001.

We applied the same modeling strategy to analyzing commenting, sharing, and subscribing behaviors, as well as adding a video to playlists. In the analyses of comments and shares, the baseline models included only the control variable, time elapsed since video upload. The second models considered video length, which did not explain additional and distinct variance above and beyond the baseline model (for comments: *χ*^*2*^ = 1.97, *df* = 1, *p* = .16, AIC increase by 0.04 from 2255.91 to 2255.95, BIC increase by 3.4 from 2265.95 to 2269.33; for shares: *χ*^*2*^ = .17, *df* = 1, *p* = .68, AIC increase by 1.8 from 2810.31 to 2812.15, BIC increase by 5.2 from 2820.35 to 2825.54). The third and final models accounted for social endorsement cues and significantly improved the second models (for comments: *χ*^*2*^ = 249.41, *df* = 3, *p* < .001, AIC decrease by 243.4 from 2255.95 to 2012.53, BIC decrease by 233.4 from 2269.33 to 2035.96; for shares: *χ*^*2*^ = 322.17, *df* = 4, *p* < .001, AIC decrease by 314.2 from 2812.15 to 2497.98, BIC decrease by 300.8 from 2825.54 to 2524.75).

Turning to our analyses of number of subscribers gained and number of playlists added in, we again included only time elapsed since video upload as the control variable in the baseline models. Next, video length was entered in the second models, which did not significantly improve the baseline models (for subscribers gained: *χ*^*2*^ = 1.39, *df* = 1, *p* = .24, AIC increase by 0.6 from 2600.75 to 2601.37, BIC increase by 4 from 2610.79 to 2614.75; for playlists added in: *χ*^*2*^ = 1.98, *df* = 1, *p* = .16, AIC increase by 0.03 from 3069.40 to 3069.43, BIC increase by 3.4 from 3079.45 to 3082.82). The third models incorporated social endorsement cues including likes, dislikes, comments and views and explained a significant amount of additional variance above and beyond the second models (for subscribers gained: *χ*^*2*^ = 382.56, *df* = 4, *p* = < .001, AIC decrease by 374.6 from 2601.37 to 2226.81, BIC decrease by 361.2 from 2614.75 to 2253.58; for playlists added in: *χ*^*2*^ = 349.74, *df* = 4, *p* < .001, AIC decrease by 341.7 from 3069.43 to 2727.69, BIC decrease by 328.4 from 3082.82 to 2754.47).

Time elapsed since a video was uploaded was significantly positively related to greater number of views (*B* = .03, *p* < .001), comments (*B* = .03, *p* < .001), and subscribers gained (*B* = .06, *p* < .001), and negatively related to number of shares (*B* = -.01, *p* < .001) and playlists a video was added in (*B* = -.02, *p* < .001). Exponentiating these regression coefficients, we obtained the incident rate ratios for views (*IRR* = 1.03), comments (*IRR* = 1.03), shares (*IRR* = .99), subscribers (*IRR* = 1.06), and playlists (*IRR* = .98). As the number of days a video has been posted online increases by thirty, the incident rates that the video gets views, comments, and subscribers increase on average by 3.43%, 2.77%, and 5.72%, respectively, with everything else held constant. The incident rates for getting shared and added in playlists decrease on average by 1.47% and 1.79%, respectively. Turning to video characteristics, video length was significantly and negatively associated with views (*B* = -.17, *p* < .001). That is, the longer a video is, the fewer views it will generate. Incident rate ratios for views (*IRR* = .84) was obtained by exponentiating the corresponding regression coefficient. With every one-minute increase in video length, the incident rate of a video getting viewed decreases on average by 15.85%, when everything else is held constant.

Social endorsement cues were indeed related to user engagement with science videos. Our results suggested that videos receiving endorsement in the form of likes was positively related to all five types of user engagement being examined––––viewing (*B* = 2.42, *p* < .001), commenting (*B* = 1.81, *p* < .001), sharing (*B* = 1.22, *p* < .001), subscribing (*B* = 2.35, *p* < .001), and adding to playlists (*B* = 1.05, *p* < .001). Moreover, with the incident rate ratios obtained from exponentiating the corresponding negative binomial regression coefficients for viewing (*IRR* = 11.20), commenting (*IRR* = 6.14), sharing (*IRR* = 3.39), subscribing (*IRR* = 10.47), and adding to playlists (*IRR* = 2.84), respectively, we found that a ten-fold increase in likes received by a video was associated with an average increase in the incident rates of getting viewed, commented, shared, subscribed, and added in playlists by 1020.12%, 513.65%, 239.28%, 947.46%, and 184.48%, respectively.

Finally, videos receiving dislikes was positively related to getting user comments (*B* = .51, *p* < .001) but negatively associated with attracting new subscribers (*B* = -.50, *p* < .001). A ten-fold increase in dislikes was associated with an average increase in the incident rate of getting commented by 66.19% (*IRR* = 1.66) and an average decrease in the incident rate of getting subscribers by 39.45% (*IRR* = .61). Receiving comments was positively related to a video being added to playlists (*B* = .51, *p* < .001), as a ten-fold increase in comments was associated with an average increase in the incident rate of being added to playlists by 66.67% (*IRR* = 1.67). Videos receiving views was also positively related to getting shared (*B* = .88, *p* < .001), subscribed (*B* = .81, *p* < .001), and being added in playlists (*B* = .59, *p* < .001), with a ten-fold increase in views being associated with an average increase in the incident rates of getting shared (*IRR* = 2.40), subscribed (*IRR* = 2.25), and being added in playlists (*IRR* = 1.80) by 140.47%, 125.04%, and 79.55%, respectively.

We applied hierarchical OLS regression to analyzing average view duration and average percentage viewed (Table 6). Time elapsed since video uploaded was unrelated to average view duration and average percentage viewed. Users watched longer videos for greater durations (*β* = .82, *p* < .001) but smaller percentages (*β* = -.48, *p* < .001) on average. In terms of social endorsement cues, videos with more likes were on average viewed for longer time (*β* = .24, *p* < .01) and greater proportions (*β* = .55, *p* < .001). In contrast, videos with more dislikes were watched for shorter durations (*β* = -.24, *p* < .001) and smaller proportions (*β* = -.43, *p* < .001) on average. Videos with more comments were watched on average for greater durations (*β* = .16, *p* < .05) and proportions (*β* = .29, *p* < .05). Lastly, videos that received more views were watched on average for shorter time (*β* = -.22, *p* < .01) and smaller percentages (*β* = -.55, *p* < .001).

**Table 6:** Hierarchical OLS regression models predicting science video average view duration and average percentage viewed (*N* = 210).

	Average View Duration		Average Percentage Viewed	
	Before entry	Final *β*	Before entry	Final *β*
*Control variable*				
Time elapsed since upload	-.27***	.01	.06	.10
Incremental *R*^*2*^ (%)	7.0***		.4	
*Video characteristics*				
Video length	.87***	.82***	-.38***	-.48***
Incremental *R*^*2*^ (%)	71.0***		13.3***	
*Social endorsement*				
Likes (log transformed)	.01	.24**	.04	.55***
Dislikes (log transformed)	-.11***	-.24***	-.19**	-.43***
Comments (log transformed)	.02	.16*	.05	.29*
Views (log transformed)	-.05	-.22**	-.12	-.55***
Incremental *R*^*2*^ (%)	3.9***		16.0***	
Total Adjusted *R*^*2*^ (%)	81.4		27.6	

Significance key:

*p < .05

**p < .01

***p < .001.

## Discussion

While video characteristics that are not primarily content-based play an important role in influencing the ranking of YouTube videos and how users respond to them, they have received less scholarly attention [18]. In addition, research that examines user engagement on YouTube is largely restricted to click- and comment-based activities while ignoring other meaningful forms of engagement activities, possibly due to limitations in data access. To better inform the practice of science communication through YouTube, our study addresses these gaps in empirical research by using a unique, comprehensive set of YouTube Analytics data from the American Chemical Society’s featured science communication and outreach channel *Reactions*. The objective of this study is to understand variations in user engagement with online science videos.

To this end, we specifically examined how video characteristics and social endorsement cues embedded in the YouTube environment explained such variations in user engagement. Although it is intuitive to speculate that gaining social endorsement such as likes will increase a video’s chances of receiving greater engagement from users, it is still up to empirical investigations to find out to what extent such social cues matter. To our knowledge, our study is the first one to have quantified the information on the interrelationships among video length, cues of social endorsement and various user engagement measures. Moreover, we present key demographic information and audience characteristics of *Reactions* toward an understanding of the makeup of audiences for YouTube science videos more generally.

Before discussing the implications of our findings for effective science outreach and public engagement through YouTube videos, it is important to note several potential limiting factors related to the interpretation of our results. First, our data came from users who were likely already engaged to some extent with science videos from the *Reactions* channel. Thus, they do not tell us much about how to engage publics that are less interested in science overall. This finding resonates with research on audiences for other types of science media. For example, blog readers were found to be sophisticated science consumers who already possessed high levels of scientific knowledge [61]. Nevertheless, other researchers have suggested that YouTube might be a platform where individuals who do not have great interest in science encounter science topics [12]. Future research should investigate how to reach potential audiences who do not already have a vested interest in science using YouTube videos or other social and entertainment media.

Second, even though video content factors were not the main focus of our analysis, they are nonetheless relevant aspects to consider when exploring user engagement. Previous research discovered that user-generated science videos were more popular than professionally produced videos on YouTube and that having consistent science communicators deliver the content might increase video popularity [21]. Use of authoritative spokespersons (e.g., politicians, scientists) rather than anonymous narrators in presenting controversial scientific topics in documentary films appeared to increase viewer engagement with the content [62]. In addition, experimental evidence showed that viewers reported greater interest and perceived relevance concerning chemistry when viewing chemistry content that focused on applications of science in people’s everyday life [63]. While we focused on factors that are not primarily content-related in this study, preliminary inspections of the titles and thumbnails of the five most and least viewed, shared, commented, liked, and disliked videos in our sample revealed that shorter and sometimes “clickbaity” titles (e.g., *Why Do Dogs Smell Each Other’s Butts*; *Is it OK to Pee in the Ocean*) helped boost viewership and engagement, perhaps because such video titles pointed to the entertainment orientation of the YouTube platform [64]. Short, concise video titles seemed to attract greater user engagement, since almost all of the least popular videos in our sample had longer titles, which were inevitably truncated on the video list page and involved displaying ellipses for omitted words in the title.

With these considerations in mind, our findings have important theoretical and practical implications for science communication research and outreach. Overall, science videos on the *Reactions* channel are doing well in reaching and engaging YouTube audiences. *Reactions* has more than 25 million views and 264,781 subscribers. The average number of views of a *Reactions* video is 122,574 and the median number of views is 48,203. To put these numbers into perspective, the median number of views for a YouTube video is 89 [65]. An average YouTube science and technology video has approximately 6,638 views [66] and an average YouTube channel has approximately 199 subscribers and 43,000 views [67]. Demographics (i.e., age and gender) of *Reactions* viewers roughly resemble that of YouTube users in general, with the 18–44 age group and males being more active and engaged. This echoes past research in that males are found to be more active audiences for online science media [1,68]. However, the audiences for YouTube tend to be younger compared with users of other types of online science media such as science blogs [61].

We found that older users (55 years old and above) do not necessarily spend more time watching each individual video than younger users but they somehow manage to watch each video in its greater entirety. It therefore seems plausible that older users tend to either watch shorter videos or to fast forward and/or speed up while watching, although empirical evidence directly supporting this speculation is lacking. Future research is needed to clarify these dynamics. In addition, the finding that female audiences are less active and engaged with science videos on YouTube may be explained in part by the fact that there is a lack of female representation in YouTube science videos. Female communicators are conspicuously absent from Science, Technology, Engineering, and Mathematics-themed video content on YouTube [30,69]. Addressing this gender gap in production of online science videos may be a necessary step for building a more inclusive, healthier YouTube science community.

Our findings reveal that whereas the majority of views of the *Reactions* videos come from non-subscribers, indicating the substantive reach of the channel beyond its subscribers, subscribers are overall more engaged with the videos. Subscribers generate approximately three times more shares and thirteen times more likes per view of video than non-subscribers. Subscribers also watch for longer time and greater proportion per view of video compared with non-subscribers. Although there appears to be a correlation between subscription status and engagement as subscribers are more engaged with YouTube science videos, it is impossible to pinpoint the exact causal mechanisms with our data. Nonetheless, prior research may shed some light on these underlying processes. Once subscribing to a channel, users will receive notifications when the channel publishes any new videos and they will be continuously exposed to those videos in their subscriptions feed [70]; therefore, subscribers have greater chances to engage with videos from the channel than non-subscribers. In addition, the channel subscription feature of YouTube fosters a communal feeling [71] and provides a vehicle for social connection and interaction with others within the online community [72], which may stimulate deeper levels of engagement. Taken together, empirical evidence suggests that science communicators may benefit from promoting subscriptions as it appears to be an effective pathway to increasing user engagement with YouTube science videos.

In addition, our findings show that computers and mobile phones are the top two access devices that generate the largest number of views. YouTube’s internal data have also confirmed that mobile devices are a major source of views, accounting for over 70% of total watch time [73]. More importantly, viewers who watch *Reactions* videos through mobile phones exhibit the highest levels of social viewing behaviors such as liking and sharing videos compared with all other types of access devices. This pattern holds even after taking into account total number of views. Prior research on college student millennial samples has similarly shown that watching YouTube videos on mobile phones is linked to higher frequency of video sharing, possibly due to the ease of sharing videos on social media apps on mobile phones [74]. Moreover, mobile devices are often used to fill the interstices during daily routines [75]. During such interstitial time, users may be more willing to communicate with peers by instantly sharing online videos [74]. These findings indicate that it may be beneficial for science communicators to create video content suited to mobile phone access as they attempt to engage wider publics using science videos.

While previous research found that longer videos were more likely to be recommended by YouTube than shorter videos [41,42], we found mixed evidence regarding the relationship between science video length and user engagement. Although on average users spent more time watching longer science videos, this might simply be an artifact of video length without necessarily reflecting quality engagement. In fact, longer science videos were watched for smaller proportions and received fewer views on average. More specifically, every one-minute increase in video length was associated with a decrease in view count by one-sixth with everything else kept constant. Users may be less likely to engage with longer videos given their limited attention spans and heightened expectations in an increasingly competitive media environment [76]. Our findings seem to support the notion that science communicators will likely benefit from producing shorter rather than longer videos. The differences between our findings regarding the effect of video length and previous work may be explained by differences in the nature of our dependent variables. Prior work focuses on video recommendation as the outcome shaped by video length, whereas we explore how video length may account for variations in user engagement. It is likely that video recommendation is not only determined by the level of user engagement, but also a function of other factors such as users’ network characteristics and other video content factors [40,77] not observed here.

Consistent with what prior research suggests, we found that overall social endorsement cues were indeed positively related to user engagement with science videos on YouTube, with a few exceptions. Among all social cues, likes appeared to have the strongest association with all seven types of user engagement—not only instantaneous and effortless click-based engagement such as viewing and sharing, but also more effortful and long-term engagement such as commenting, subscribing, and adding to playlists and attention-based engagement such as viewing a video for longer and viewing a video in its entirety. Unlike other social endorsement cues such as comment and view counts, likes explicitly indicate users’ favorable evaluation and/or appreciation of video content [23] and therefore may play a more influential role in convincing prospective viewers that the video content is worth engaging with. The findings that videos with more likes on average received more views and were watched for longer durations also echo previous research findings that online information accompanied by a higher number of likes was chosen by users more often and earlier and was read for longer [53]. This demonstrates that there is value in identifying ways to increase social endorsement on videos, especially in the form of likes, as this social cue has the greatest potential of stimulating other viewers’ subsequent engagement behaviors.

As expected, videos with more dislikes received less user engagement in terms of subscribers gained, average view duration, and average percentage viewed. However, greater dislikes were associated with more comments received. The causal mechanism may go two ways: on the one hand, a high number of dislikes may indicate that a video contains controversial content, which may consequently elicit more user comments [78] because users feel a strong emotional urge to express their opinion on the issue [79,80] and because controversy increases interest [81]; on the other hand, research on the “nasty effect” suggests that uncivil online comments can polarize audience perceptions of the risk of emerging science and technologies among certain groups [82], thus potentially making some users disfavor aspects of the video content.

Finally, although videos with higher view counts were also shared and added to playlists more often and attracted more subscribers, they received lower attention-based user engagement in terms of both average view duration and average percentage viewed. While there is little empirical research evidence that can easily reconcile these findings, we speculate that the most highly viewed videos enticed a larger audience base than the channel’s usual suspects, due to peripheral factors such as “clickbaity” video titles and descriptions. In other words, users might be attracted to those videos hoping for something else than what the videos were really about, and once they found out about it, they stopped watching. However, more research is needed to unravel the relationship between video view count and the attention-based user engagement measures before any final conclusions can be drawn. Moreover, our findings point out that science communicators will likely run into competing demands when optimizing their public engagement strategies. When this occurs, communicators should be clear about which goals to prioritize given that different goals may determine different courses of action.

More importantly, science communicators should pay attention to how increasing user engagement fits into the broader goals of public communication of science. The online measures of user engagement provided by YouTube are an imperfect indicator of the degree to which science video consumption leads to the various outcomes that effective science communication seeks to accomplish, including sharing the findings and excitement of science, growing appreciation for science as a useful way of making sense of the world, increasing knowledge and understanding of science, influencing people’s opinions, behavior and policy preferences, and engaging with diverse perspectives about science held by different publics in solving societal problems [13]. Increasing user engagement is a necessary condition rather than a substitute for those other goals of public communication of science. As science communication today takes place in an increasingly complex and high-choice media environment where “many voices are competing for the attention of various audiences on all topics, including science” [13], increasing user engagement helps science videos cut through the online noise and reach their intended audiences and is therefore instrumental to effective science communication. However, communicators should not lose sight of the more important, overarching goals of science communication while seeking to optimize user engagement. Specifically, practitioners should not lower the quality or truthfulness of their content in exchange for, say, more clicks, as this could ultimately harm the goals of public communication of science.

In sum, we believe our findings illustrate the importance of attending to not just video content factors but also factors that are not primarily content-related when understanding various user engagement activities with online science videos. Using videos for public engagement and science outreach has gained increased popularity in practice and has ample room for growth in research [11]. Documentaries and other science video programs have become the second largest source of science news for Americans and the second most trusted source of science facts [11]. To guide sound practice of communicating science through YouTube, future research should look into the complex interplay among video factors that are both content-related and content-independent. While such dynamic can be complicated, we hope our study may contribute to future endeavors to disentangle and interpret these processes more clearly.

## Supporting information

S1 File Microsoft excel dataset file of *Reactions* YouTube videos (titled [dataset_reactions_videos.xlsx]). (XLSX)
